# Refractory Biliary Catheter Leak Corrected by a Hybrid Closed Loop Catheter-Pump System

**DOI:** 10.1155/2021/6677500

**Published:** 2021-03-09

**Authors:** Ravi Murthy, Varun Rachakonda, Juri Bassuner

**Affiliations:** ^1^Departments of Interventional Radiology & Investigational Cancer Therapeutics, M.D. Anderson Cancer Center, The University of Texas at Houston, Houston, Texas 77030, USA; ^2^Houston Methodist Hospital, Department of Radiology, 6565 Fannin St., Houston, TX 77030, USA; ^3^Integrated IR/DR Residency, PGY-4, Saint Louis University School of Medicine, 3635 Vista Ave., St. Louis, MO 63110, USA

## Abstract

The development of inoperable biliary obstruction in patients with liver, biliary, and pancreatic neoplasia is commonplace particularly in the advanced stages of these diseases. Under these circumstances, restoring bile flow to the gut is paramount in reestablishing homeostasis. Hitherto, this has been achieved by utilizing passive, gravity-dependent bilioenteric conduits with the use of perforated plastic catheters or metallic stents inserted either in a percutaneous transhepatic fashion or via endoscopic techniques. A frequent untoward event of biliary decompression utilizing percutaneous transhepatic catheters (PTC) is the development of pain, cholangitis, hyperbilirubinemia, or pericatheter bile leak due to the suboptimal normalization of bile flow. In some instances, the etiology of PTC malfunction can be correctly ascribed to catheter malposition and/or catheter lumen obstruction; however, in the majority, it remains radiographically occult on transcatheter cholangiography—the “gold standard.” Regardless of findings, the management remains fluoroscopic repositioning or exchanges for larger diameter catheters to attempt to seal the pericatheter potential space and prevent bile seepage. Unfortunately, these maneuvers are met with limited and unpredictable levels of success. We present the successful management of an instance of recalcitrant external pericatheter bile leak mitigated by employing a hybrid closed loop biliary catheter-pump system by employing an assortment of FDA approved off-the-shelf medical devices.

## 1. Case Presentation

This study was conducted with Institutional Review Board approval and complied with the Health Insurance Portability and Accountability Act.

**Figure 1:**
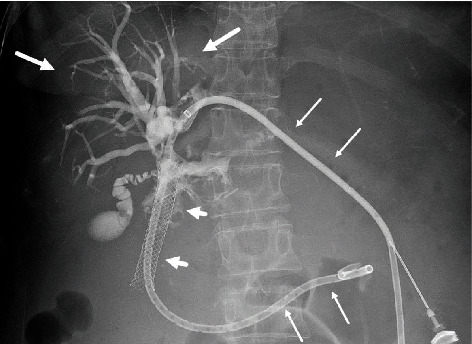
Contrast cholangiography via internal-external biliary drainage catheter (long arrows) demonstrates intrahepatic biliary ductal dilatation (large arrows). There is an occluded metal stent (arrowheads) with no flow of contrast beyond this point into the bowel.

A 38-year-old female with an unresectable, locally advanced, intraductal papillary pancreatic mucinous neoplasm and an indwelling endoscopic metal stent for biliary obstruction developed bouts of cholangitis. The duodenal involvement from the pancreatic cancer rendered repeat intervention via the ERCP route difficult and therefore was not entertained, and the patient was referred for salvage percutaneous transhepatic biliary catheter decompression. Utilizing the combination of ultrasound-guided cholangiopuncture and fluoroscopic assistance, biliary access was achieved via the left lobe of the liver, and a 10-French Dawson-Mueller (Cook Medical, Bloomington, IN) Cope retention loop catheter was reformed within the common hepatic duct and connected to external gravity drainage. Copious pericatheter bile leak ensued. Transcatheter cholangiography failed to elucidate the etiology of the pericatheter leak. In accordance with our institutional clinical practice, the catheter was empirically “upsized” to 12, 14, and 16 Fr diameter external (Mac-Loc, Cook Medical, Bloomington, IN) catheters in separate but closely spaced sessions without relief. The penultimate maneuver, which was expected to be unsuccessful *a priori*, involved insertion of a 12 Fr Ring internal-external biliary catheter (Cook Medical, Bloomington, IN) (Figure 1).

Conventional maneuvers had failed to remedy the profuse external pericatheter leaking. The underlying problem was recognized as one of hydrodynamics. We therefore envisioned constructing a device that possessed an active propulsive mechanism for bile movement coupled with a dual lumen catheter system to facilitate bile movement separately from the intrahepatic bile ducts and gut as a possible remedy.

**Figure 2:**
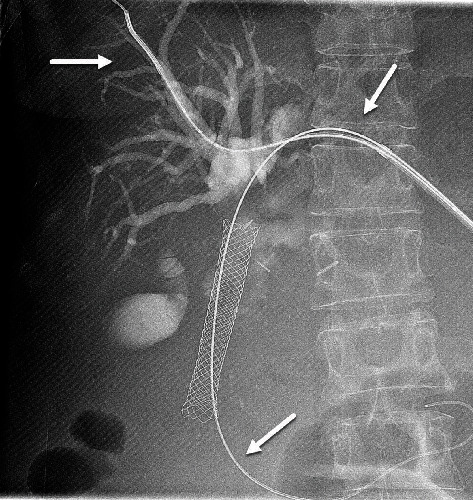
Exchange of internal external biliary drainage catheter for vascular sheath. Insertion of 2 guidewires, one each in the intrahepatic biliary radicles and one in the duodenum (long arrows).

A 16-French, 32 cm Medcomp® Split Cath® dual lumen hemodialysis catheter (Medical Components, Inc., Harleysville, PA), which possesses the inherent ability to variably separate apart the catheter lumens, was identified as meeting requisite criteria. Following removal of the indwelling biliary catheter under fluoroscopy, two Super Stiff Amplatz wires (Boston Scientific, Natick, MA) were negotiated separately into the intrahepatic right lobe ducts and duodenum via an 8 Fr vascular sheath. After measuring the intended length of the intrabiliary catheter component, one catheter moiety was manually trimmed and side holes were cut. The Dacron cuff was removed, and the catheter lumens were then simultaneously yet individually advanced over these two wires such that the efferent intrabiliary limb with additional side holes was draped over confluence of the right and left hepatic ducts, and the afferent enteric limb was advanced into the duodenum (Figure 2). The corresponding external limbs were connected to a Jackson-Pratt bulb (JP bulb) with luer lock extensions. As intended, the bulb was squeezed to create suction, and the stopper was closed to maintain the suction.

**Figure 3:**
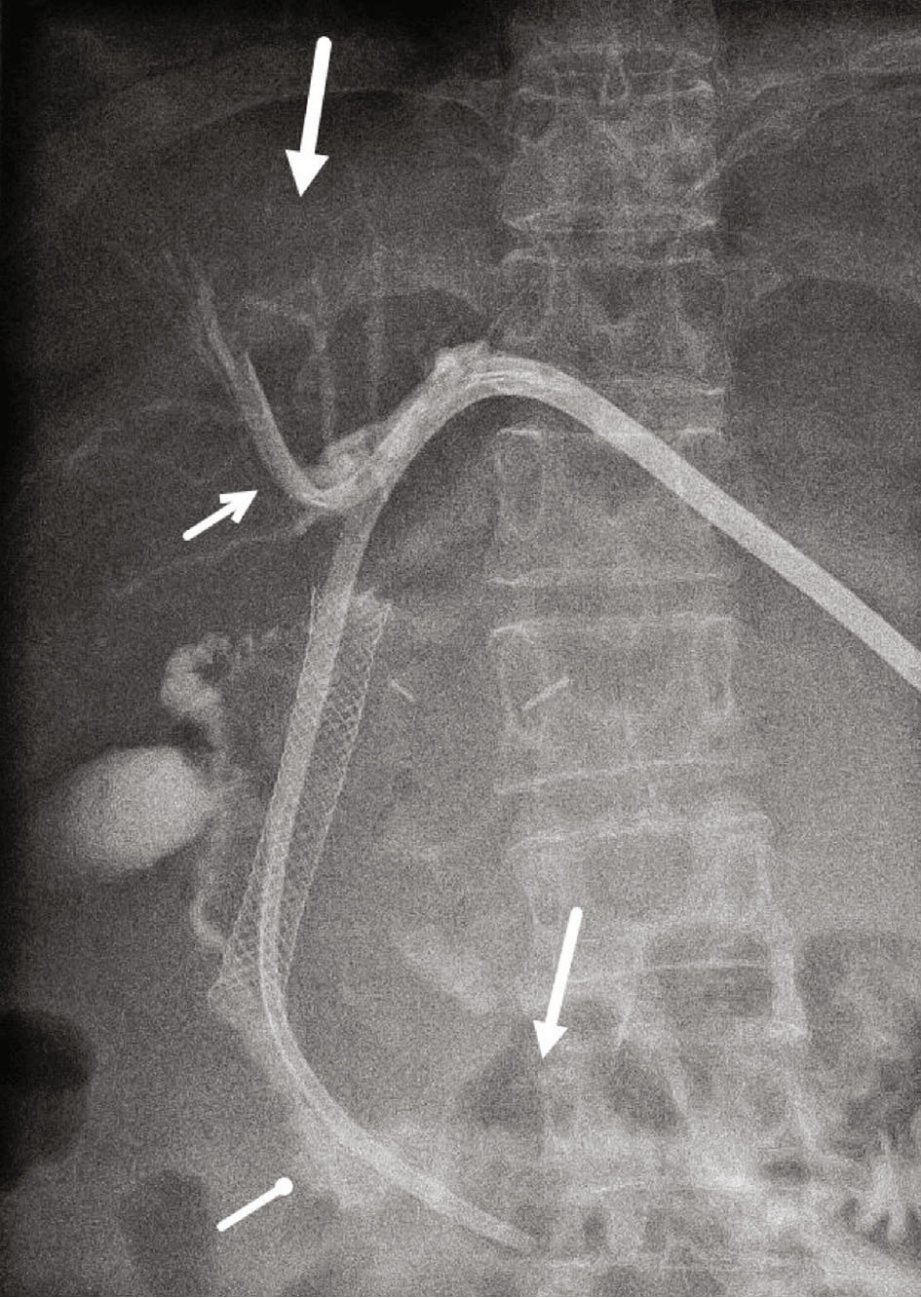
Contrast cholangiogram via dual, split lumen catheter. The efferent catheter limb drapes the biliary confluence (small arrow), and the afferent catheter limb is in the duodenal lumen (round tip arrow). Normal caliber of the intrahepatic radicles (large arrow) with contrast within the duodenum (long arrow) is noted indicating biliary decompression and bile deposited in the bowel.

Radiographic confirmation of the functionality of the system was obtained by injecting contrast through the efferent, intrabiliary lumen and promptly connecting the primed JP bulb. Once aspirated bile (mixed with contrast) was visualized in the bulb, the bulb was squeezed, resulting in contrast being ejected through the efferent limb into the duodenum (Figure 3).

**Figure 4:**
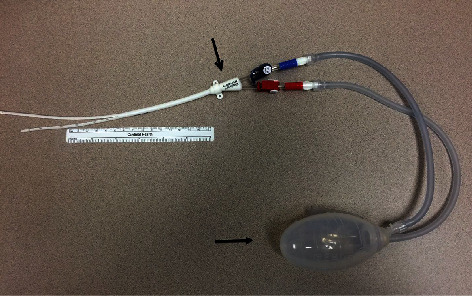
Hybrid closed loop catheter system; table mock-up. Dual channel, separable lumen hemodialysis catheter (open arrowhead) with additional side holes in the shorter venous limb connected to a JP bulb suction bulb (closed arrowhead).

A table mock-up of the catheter was utilized to educate the primary caretaker who was instructed to decompress the bulb when it filled (Figure 4). The patients' leaking decreased dramatically, and she was discharged home following at-best palliation of her other medical issues until her demise 2 months later.

## 2. Discussion

Herein, we describe an application of existing off-label device technologies to mitigate persistent pericatheter bile leak with the added benefit of allowing replenishment of bile to the gut. This rudimentary hybrid closed loop catheter-pump system allowed palliation of her bile leaking and hospital discharge.

In clinical practice, the underlying principles of the PTC technique and equipment have remained unchanged over 5 decades since the initial descriptions of percutaneous biliary catheter drainage [1–4]. Technical success remains high, but adverse events such as bile loss, leaking, and cholangitis have remained a major source of morbidity [5–7]. Bile flows within the PTC along small pressure gradients [8]. Dehydration and bile contact with enteric content further alters bile viscosity [9]. Several investigators have attempted to augment the transfer of bile to the gut by increasing the catheter diameter [10], external biliary catheter crosslinking to an internal biliary catheter [11], or a jejunostomy via a peritoneovenous shunt pump [12], anchoring with a balloon tip catheter to prevent migration [13], and creation of additional side holes [14] with variable and unreliable levels of efficacy.

Hepatobiliarypancreatic cancers are in an exclusive group of cancers whose incidence is increasing, and mortality is unchanged. [15, 16] Their lethality is largely due to the consequences of biliary obstruction. Furthermore, as therapeutic and supportive cancer control measures improve survival, the expected increasing prevalence of hepatic metastases will also result in higher numbers of patients with biliary obstruction and the need for biliary decompression. This implies a need for evaluating for improvements in PTC-related technologies, as our approach has shown, that may assist in providing the required support for the expected increase in demand for these and allied services.

## Data Availability

Data is available within the secure institutional electronic medical record in which the corresponding author is faculty.
